# Case Report: Why Sleep and Dream Related Psychological Treatments, Such as Sleepcoaching (According to Holzinger&Klösch) and CBT-I Should Be Implemented in Treatment Concepts in the Public Health System—Description of the Nightmare Treatment Process in the Context of PTSD

**DOI:** 10.3389/fpsyg.2021.733911

**Published:** 2021-10-25

**Authors:** Brigitte Holzinger, Franziska Nierwetberg, Gerhard Klösch

**Affiliations:** ^1^Institute for Consciousness and Dream Research, Vienna, Austria; ^2^Certificate Program Sleep Coaching, Medical University of Vienna, Vienna, Austria; ^3^Department of Neurology, Medical University of Vienna, Vienna, Austria

**Keywords:** nightmares, sleepcoaching, PTSD, lucid dreaming, sleep, dreams, CBT-I, public health

## Abstract

In this case report, we explain the story of a woman diagnosed with severe PTSD, suffering from recurrent nightmares involving a traumatizing event. She participated in 6 week lucid dreaming training to help her reduce her nightmare frequency. Our descriptions include her dream reports as well as the results of the psychological assessment conducted. In only 6 weeks, she was able to begin to change her dream plots and to improve several of the psychological measures. In this case, we stated that paying more attention to sleep and, especially nightmares, not only in patients with PTSD, should be standard in treatment processes for psychiatric disorders. We, therefore, underpin our case with literature that explains the benefits of treatments, specifically for sleep problems that do not involve medication.

## Introduction

This case study is a part of a sample of “cases” we had the honor to collect in the project “Cognitions in Sleep—Lucid Dreaming as an Intervention for Nightmares in Patients with PTSD,” published 2020 in Frontiers of Psychology (Holzinger et al., 2020). In the context of a nightmare treatment evaluation research project in patients with posttraumatic stress disorder (PTSD) in 2009 at the inpatient rehabilitation clinic in Europe (we do not state specific countries in this article to provide additional protection of any identities), we had the opportunity to offer a lucid dreaming (LD) program, which we also call Cognition in Sleep (CIS) to inpatients with PTSD. LD is characterized by a person being aware that he/she is dreaming and by voluntary control over the dream plot (Holzinger, 2008). Lucid dreaming as a treatment for nightmares is part of our holistic approach called sleepcoaching, which is based on Gestalt Therapy (Holzinger and Klösch, 2013; Holzinger et al., 2019a,b). Sleepcoaching consists of four elements: sleep education and sleep hygiene, cognitive behavioral therapy for insomnia (CBT-I), relaxation techniques, including medical hypnosis, and dream work, including LD techniques. During this program, our focus, of course, was on LD techniques. Nevertheless, it did incorporate aspects of sleep education and sleep hygiene.

In patients with PTSD, addiction problems and multiple comorbidities were treated in a 3 month psychotherapeutic program. Most of the patients were under excessive medication, as the risk of suicide was considered very high. PTSD is a psychiatric disorder emerging after a traumatic event or situation. Those events or situations evoke strong feelings of desperation in the affected person. Typical symptoms of PTSD are recurrent re-experiencing of an event, intruding memories of the event, dreams, nightmares, and a lingering feeling of numbness and emotional stupor. In addition, symptoms similar to depressive ones, like joylessness, reduced activity, and carelessness, may occur. If those feelings remain over a long time, this can also lead to suicidal thoughts (World Health Organization, 2017). The distress of reliving the traumatic events not only by flashbacks in the daytime but also in nightmares during sleep causes a great extent of psychological strain. Those disturbing dreams and nightmares are the core symptom of PTSD and are very common, as the prevalence of nightmares is 60%, which is the highest among mental health disturbances. The prevalence of PTSD is about 8–9% in the general population (Krakow et al., 2002). Nightmares among patients with PTSD tend to occur in two predominant forms:

1. Intrusive replicative nightmares that reenact parts of the trauma and cause difficulties initiating or maintaining sleep.
2. A form of nightmares corresponding to the re-experiencing and hyperarousal symptom cluster defining PTSD.

(Levin and Nielsen, 2007).

Nightmares, in general, are defined as dreams being “extended and extremely dysphoric,” and that “usually involves efforts to avoid threats to survival, security, or physical integrity.” They usually occur during REM sleep and tend to wake up the dreaming subject, remembering the dream vividly (American Academy of Sleep Medicine, 2014).

The case reported in the following sections displays how dream work, including LD training, was able to promote improvements concerning the nightmare and other symptoms in the reported PTSD case very quickly. Furthermore, it represents severe and multiple traumatizations, which conditioned a long history of PTSD with various sleep problems and other comorbidities in the patient.

## Case Report of a Female Participant in the Add-On Lucid Dreaming Training Program

The LD program was conducted as an add-on treatment to the regular trauma treatment. The program was offered weekly for 1 h in a group setting for a maximum of eight participants. All patients with PTSD of the rehabilitation center were informed about that service and voluntarily signed up. The ultimate goal of the program was to overcome nightmares by the use of LD techniques (cognition in sleep).

The program offered general information about sleep, sleep hygiene, and also on how to keep a sleep and dream diary, as this was the documentation method to keep track of the dreams of the patients. The core target of the program, to improve nightmare complaints by means of LD, was built on knowledge about dreams, nightmares, and LD and what they might do for us.

From the pool of patients participating in our program, one stuck out. We report the case of a female inpatient at a rehabilitation clinic; she experienced multiple traumas from childhood, for which she received treatment for PTSD. Her process is also described in *Lucid Dreaming: New Perspectives on Consciousness in Sleep* (Hurd and Bulkeley, 2014).

Mrs. L. (the initial of her name is altered) was traumatized multiple times throughout her life. She was sexually abused by her grandfather several times and was raped by a group of teenagers when she was 13 years old. She was unhappily married and divorced after 3 years. Her ex-husband did not accept this step and stalked her with a gun, and, by holding up the gun to her head, forced her to come back to him. During her marriage and, for some time after, she was living in a different country, but after those incidents, she moved back to where she was born. Shortly after moving, she got involved in a severe car accident, from which she had to recover for several months forcing her into sick leave for that time. After recovering, she started to work as a travel agent in a workspace separated by glass walls. Due to the hectic nature of this demanding job, she oversaw that glass wall and injuring herself severely by a craniocerebral trauma.

All those incidents reinforced insomnia symptoms, characterized by problems of falling and maintaining sleep associated with frequent nightmares. The nightmares, which occurred since childhood, were often recurrent, involving her ex-husband stalking and threatening her, occurring several times a week. As a consequence of these nightmares, Mrs. L. developed aversive feelings toward sleep, especially after nightmare episodes.

Mrs. L., as she had experienced several traumatizing situations in her life, developed a quite pronounced PTSD, which had been treated with psychopharmaceuticals (mornings: Duloxetine, 60 mg, evenings: Trazodone, 15 mg; Quetiapine, 25 mg). Additionally, as she was an inpatient at a rehabilitation clinic, she received the standard psychotherapeutic treatment for PTSD and traumatization in this clinic, which is single and group psychotherapy. She did not receive treatments targeting her sleep problems, except the medication listed.

Before entering the LD program, Mrs. L. had undergone excessive psychometric testing. The results of the *Symptom Checklist-90-R* (*SCL-90-R*: Derogatis, 1977) suggested that Mrs. L. had severe somatic complaints, high scores in anxiety, phobic fear, obsessive-compulsive disorder, and depression. The *Self-rating Anxiety Scale* (*SAS*: Zung, 1971) was conducted and confirmed an anxiety disorder; the Impact of Event Scale-assessed PTSD and the *Self-rating Depression Scale* (*SDS*: Zung, 1965) confirmed high scores in depression. In addition, sleep disorders were also evaluated. The *Epworth Sleepiness Scale* (*ESS:* Johns, 1991) yielded pathological daytime sleepiness; the *International Restless Legs Syndrome Study Group rating scale for restless legs syndrome* (*IRLSSG*: Walters et al., 2003) revealed the presence of restless leg syndrome. This tool is assessing the severity of restless leg syndrome rather than diagnosing it, but, as it was the only measurement used and when looking at the results, we came to the conclusion that Mrs. L did, indeed, suffer from restless leg syndrome. Furthermore, the *Schlaffragebogen-B* (*Sf-B*: Görtelmeyer, 1986) indicated severe insomnia symptoms. Her overall sleep quality as measured by the *Pittsburgh Sleep Quality Index* (*PSQI*: Buysse et al., 1989) was 14, indicating chronic sleep disorders. Mrs. L also suffered from nightmares several times a week and thought about them often during the day. Consequently, her overall quality of life, measured by the *Quality of Life Index* (*QLI*: Ferrans and Powers, 1985), was very low (a total score of 3.7 in the QLI). The extent of her traumatization was measured by the *PTSD Symptom Scale* (*PSS*: Foa et al., 1993), containing three subscales, namely Intrusion, which measures the re-experience of the traumatic event; Avoidance; and Hyperarousal. Additionally, the *Impact of Event Scale* (*IES:* Horowitz et al., 1979) was conducted. Both measures indicated severe traumatization of Mrs. L. The measurements listed above and dream reports were collected at the end of the training program and 6 weeks after the end, as a follow-up assessment. Results of all time points are displayed in Table 1.

**Table 1:** Mrs. L.'s scores of psychological measures.

	**Begin**	**End**	**FU**
PSQI	14	9	16
ESS	16	15	11
IRLSSG	18	12	13
SCL-90-R	71	73	80
SAS	53	49	Missing
SDS	56	57	62
QLI	3.7	3.5	2.7
IES	−3.69	−1.12	−0.93
PSS	41	26	37
Intrusion	3.75	3.5	2.375
Avoidance	2.75	1.625	2.25
Hyperarousal	3.67	3.17	2.83

*Begin, beginning of training; End, end of the training; FU, follow-up*.

During the 6 week training program, Mrs. L reported two nightmares and two positive dreams in the first week. In the course of the LD-training program, she reported 13 dreams; 10 of them were nightmares, and, of those nightmares, four showed plot changes. In the second week, she reported three nightmares; in the third week, one positive dream; during the fourth week, one nightmare with a changed plot and one positive dream; during the fifth week, she reported three nightmares with changed plots; and during the sixth and final week of the program, there was no information about her dreams.

We started the therapy by explaining to her how she can try to learn LD. Mrs. L. was very motivated to do so and followed the suggested steps. We also discussed several ways and options that she could choose from when dreaming her nightmare to try to change it. Those strategies were developed by asking the whole training group what they could suggest and discussing the ideas in the group. It is important in our approach that solutions are not predetermined by the therapist but developed together with the clients. Examples of the resulting options were searching for a safe place in the dream or looking directly at what is happening to see what is actually scary. After only 1 week, the first moderate changes in her dream were noticeable in her dream report:

Week 1: 02.02.2009 (rated as a nightmare):

*I dreamt at around 2 am that my ex (former boyfriend whom she was separated 12 years ago) chases me by car through the city in which we used to live. He yelled: “I'll catch you and I'll kill you.” Running away, I felt bad aches in my legs and couldn't move right. This all happened at night, nevertheless I saw him and his pointed gun. He tried to shoot at me but didn't hurt me. I woke up in my bed sitting [up]*.

The next morning (week 1: 02/03/2009) she reported on the following dream:

*A colleague of my ex had seen me and told him about that. He, again, chased me through the city in a car*—*never mind where I ran*—*he found me everywhere and grinned sadistically. Shortly before he ran me down with his car, I woke up. I fell back asleep and dreamed that dream again*.

This time she woke up before something really happened. Her dreams continued to change during the course of the group training, and her dreams started to become more masked and started to be situated in different settings. She reported dreams that she did not perceive as nightmares like the following:

Week 1: 02/05/2009:

*I dreamt around 04.30 am that. I was at the dentist, he drilled a tooth, fixed it and said that I will live another 50 years with these teeth. The dental office was also a boutique for women's cloths where I wanted to buy some clothes. However, the dentist paid for everything. Another woman wanted to persuade me to start an affair with him. I refused. She said I should do that and loose my financial problems. I left laughing*.

The next week, dreams including her ex-boyfriend recurred:

Week 2: 02/09/2009:

*I dreamt at 2.30 thatI was surrounded by many cabs. In each of them I saw the sadistic grin of my ex-boyfriend (who is a cab driver). Through a loudspeaker he yelled that he will catch me and kill me, wherever I ran to, he was everywhere. Woke up around 4 am, continued dreaming. He again was in many taxis, pushed me into a dead end street, I was able to disappear into a house and woke up sweating*.

Week 2: 02/10/09:

*I dreamt at 2 am that… I was followed by me ex-boyfriend in his cab. Through the window he pointed his pistol towards me and yelled that he will kill me. After having been chased by him for a long time, I turned around and yelled that he should do it and stop torturing me. He shot at me, but didn't hit me and disappeared. I woke up sweating*.

Week 2: 02/11/2009:

*I dreamt at 2 am that … I was sitting in the cab of my ex-boyfriend, he drove at extreme high speed, I was nauseous and my head was spinning, he yelled at me that he would now kill me. Then we fell down a cliff and I woke up sweating*.

All of these three nightmares during the 2nd week included the topic of her ex-husband stalking and threatened her. Even if those dreams cannot be considered to be changed in the plot, there is still a slight shift noticeable. One time, she did manage to escape into a house; the next time, she did escape the role of the victim for a short moment yelling at him, and another time, they both fell down the cliff.

During the 3rd week, she did have some dreams not considered being nightmares and off that topic surrounding her ex-husband.

The changes of the dream plots became more pronounced in dreams about her ex-husband in the 4th week:

Week 4: 02/26/2009:

*At around 3 am I dream that my ex chases me, again by cab. I cried for help, many people came out of their houses and together we walked towards him. He disappeared*.

Week 4: 02/28/2009:

*I dreamt at 2.30 am that … I was driving through a wood with many people. We stopped at a restaurant. At once I was with a former colleague, much younger than I am and I had sex with him on a bench. The others, strangers to me did not notice apparently. Afterwards I yelled at him that I was afraid to catch an illness because his penis was deeply red. I ran away and woke up*.

The most fundamental change was that she was now looking for help and did find help. Still, this dream is a nightmare, but her helplessness has disappeared. She gained new strength that allowed her to walk toward the stalker ready to confront him instead of only running away, and he disappeared. We had previously talked about this solution for her nightmare, and, even if she did not dream lucidly, she still incorporated this novel idea in her dream. The technique to find people a safe place or trying to acquire help during a nightmare may occur during PTSD treatment because helplessness is one factor hindering patients to let go of their traumatization. After 27 days of nightmare treatment, Mrs. L. reported the following dream:

Week 5: 03/02/2009:

*At around 2:30 am I dreamt again that my ex chased me again in a cab. I ran and ran but was unable to cry for help; no sound came out; my voice had left me. He threatened me again with his gun. At some point, I was able to simply yell, “STOP.” I woke up. After I had fallen asleep again, the dream continued, I ran, stumbled and woke up*.

In this first dream, Mrs. L. was able to make a conscious decision to wake up and escape the situation, but she was not able to do the same in the subsequent dream. She kept having this dream, sometimes able to change it, sometimes not. This was her last dream report:

Week 5: 03/03/2009:

*My ex chased me in a cab. This time the pointed gun was oversized. With a sadistic grin he yelled that he will kill me. I now yelled back that he should do it. I ran towards a downhill slope, he stumbled and fell down the hill. I looked but couldn't see him anymore. The cab and he had disappeared. I woke up, this time not frightened, but somewhat relieved*.

In this dream, one could assume that she lost her fear to some extent. It was not necessary to seek help to confront her victimizer, and she did not choose to wake up. Even though this cannot be interpreted as healing, as the number of nightmares remained stable, it shows a significant change. Moreover, her anxiety and depressive symptoms had decreased. This outcome is very promising as no one expected those multiple traumas to be overcome in just 6 weeks.

Mrs. L. still reported dreams about her ex-husband, but it did not occur as often as before and it showed variations. In the 2 weeks, following the treatment, the contents of helplessness still dominated, maybe because she had to deal with her nightmares on her own again. After that, she was again able to make her ex-husband disappear, rescue herself, or could make herself wake up. Specifically, during the first week following the treatment, we got no information about her nightmares or dreams. In the second and third weeks after the program, she reported one nightmare each. During the fourth week, she reported two nightmares and, during the fifth week, one nightmare. In the sixth follow-up week, we got no information.

We believe that manipulating dreams directly is a more effective way of gaining inner strength than reframing waking fantasies in treatments.

At the end of the LD training, the diagnostic assessment had changed (Table 1). Her sleep quality improved, as, for example, she managed to shorten her sleep latency. However, she still had and remembered nightmares. Additionally, her overall quality of life improved slightly.

An LD training program for only 6 weeks is quite efficient and supportive but may have been too short to change traumatic experiences persistently. Between the end of the training program and the follow-up 6 weeks later, the dreams of the client suggest a form of relapse.

03/29/2009:

*I dreamt at 2:30 am that … my ex followed me once more again in his cab, no people, no help! I ran and ran until I stumbled and fell. He drove over me and disappeared*.

04/04/09:

*I dreamt at 2.30 and 4 am that … my ex followed me in his cab and pointed with a pistol towards me. Yelled with a horrible laugh that he will get me and kill me. I was able to slip out of the dream twice, woke up, but fell asleep again and continued the dream. The 3*^*rd*^ *time he shot around like a mad man and I woke up*.

04/09/2009:

*I dreamt at 2 am that … my ex drove in his cab towards me on the pedestrian zone and yelled hysterically that he will shoot me. He exited the car, pushed me toward the wall of a house and was about to shoot. I yelled, looked him straight in the eyes and he disappeared*.

04/10/2009:

*I dreamt around 2 am that… I drove with my ex in his cab, and he yelled that he will kill me, nevermind how. He threw me out of the car, turned and wanted to drive over me. I ran and ran and cried for help and woke up*.

04/14/2009:

*I dreamt at around 3 am that … my ex followed me again in his cab and yelled. I didn't see him much. When he pointed his pistol by the window towards me I yelled “Do it” and he disappeared*.

## Discussion

The LD program was very well-received by all participants and was very informative for both sides. One of the most impressive observations on my part was that people with severe psychological problems, including PTSD and addiction problems, also often seem to additionally and substantially suffer from sleeping problems. This indicates that a treatment specifically targeting these problems should be implemented. People coming to a clinic are usually asked at the beginning of in-patient therapy about potential sleep impairment and are then given drugs for curing sleep problems. Pharmaceutics often cause various side effects, such as drowsiness and fatigue, during the day and lead to dependencies in patients with addictions, a common comorbidity of PTSD (Friedman, 2013).

In nightmares, a negative relationship between nightmares and poor sleep quality has frequently been reported (Schredl, 2003). Having frequent nightmares often results in trying to avoid sleeping, to begin with (Rosenberg and Van Hout, 2014), and the impacts of low sleep quality have been explored in the former section. The concern is also the fact that studies have already confirmed that having a nightmare induces behavioral consequences the next day, even in healthy subjects, namely higher anxiousness, mental instability, and even physical complaints (Köthe and Pietrowsky, 2001). Furthermore, having frequent nightmares was associated with declined mental health and sleep quality as well as increased suicide risk in patients suffering from mental disorders (Lemyre et al., 2019). Especially, that nightmares predict suicide risk that is found in several studies (Tanskanen et al., 2001; Sjöström et al., 2009; Nadorff et al., 2011, 2013, 2014) is concerning and further supports the demand to take frequent nightmares as an indicator for severe mental disorders, including suicidal risk (Nadorff et al., 2011), and their individual consequences on mental health, seriously. This notion gets further supported by results that nightmares predicted repeated suicide attempts, while no other sleep problem did so (Sjöström et al., 2009), and that they also predict self-harmful thoughts and behavior in a unidirectional way (meaning that nightmares predicted those thoughts and behaviors, but those thoughts and behaviors did not predict nightmares) (Hochard et al., 2015).

The treatment of nightmares by learning LD has been the topic in a recent review (de Macêdo et al., 2019), with some promising results: decreases in nightmare frequency and reduction in the intensity of psychological distress. But the literature on this topic is still inconclusive, and further studies are needed. The present case further highlights the potential that LD has to help with nightmares.

The aim of this therapeutic approach was to provide patients with the possibility to influence their dreaming, or even allow them to control nightmares while they occur. The aspect of having control is substantial as patients with PTSD often suffer from feelings of helplessness and makes this approach even more promising for this group of patients. Neurophysiologic studies suggest that brain regions associated with self-awareness and semantic understanding are significantly more active during LD as compared with non-lucid dreams (Holzinger et al., 2006). Implementing LD in Gestalt Therapy programs for the treatment of sleep problems was found to reduce symptoms more quickly, and positive effects were even found in patients not able to achieve LD (Holzinger et al., 2020). Own studies show that treating nightmares in patients with PTSD by LD has formerly decreased anxiety and depressive symptoms but did not improve sleep, including nightmares (Holzinger et al., 2020).

Mrs. L did not achieve to learn how to dream lucidly during this very short time period of the 6 week training. Still, it had a positive impact on the distress stemming from those nightmares. After the LD training, we observed that various measures improved. And even though the number of nightmares did not decrease, the nightmare plot changed, and the nightmares were less frightening. We, therefore, concluded that LD helped with nightmares, and, furthermore, that it improved psychological health and sleep quality in general.

The benefits of the training regressed to some extent after it ended. We suggest that had been the training continued, further progress would have been visible. This gets further support by the fact that, as we spoke to Mrs. L to get her consent for the present article, she mentioned the strategies she learned in our LD training are still helpful and her nightmare problems have further improved.

Based on these findings and the effectiveness of Cognitive Behavioral Treatment for Insomnia (CBT-I) (Morin, 2015; Ballesio et al., 2018; Baglioni et al., 2020), we defined our sleepcoaching concept: Its' theoretical background is Gestalt therapy and it involves CBT-I and relaxation techniques, but is still addressing dreams and nightmares by implementing dream work and nightmare treatment (including LD training programs, particularly for PTSD patients). This inclusion of dream work and LD is the unique selling point, which distinguishes sleepcoaching from other psychotherapeutic approaches to sleep problems. We argue that sleepcoaching, as sleep problems are common comorbidity to various disorders, should be implemented in treatment plans in the public health system by default. Sleep problems and nightmares share a close relationship (Schredl et al., 2016), which gets also visible through the case of Mrs. L., as not only her nightmare symptoms but also other sleep and psychological health-related measures improved. These problems should, therefore, not be treated separately but in one holistic approach, like sleepcoaching. Patients would not only benefit from learning how to dream lucidly, which may improve their overall sleep quality but from sleepcoaching, which is a method to introduce knowledge that promotes healthy sleep habits and better sleep hygiene. Sleep hygiene education has often been proofed to improve sleep quality measures (de Sousa et al., 2007; Chen et al., 2010; O'Donnell and Driller, 2017). A review of the effectiveness of sleep hygiene education argued that sleep hygiene recommendations are most effective when individually fitted to the life of an individual, which is the case in the context of sleepcoaching (Irish et al., 2015). Another important benefit is that sleepcoaching has no unwanted side effects or interactions and, therefore, is applicable with any medication or disorder. Nevertheless, well-balanced drug treatment is sometimes supportive in combination with CBT-I and sleepcoaching by catalyzing additional improvement. Studies confirmed that several sleep measures improved after just a 2 day seminar on sleepcoaching (Holzinger et al., 2019a). This gets additional support as not only nightmares of Mrs. L. seemed to shift but other psychological problems as well. For example, her anxiety, depression, and quality of life score also improved. These changes already occurred over a time period of just 6 weeks.

As mentioned before, sleep problems are frequent comorbidity in various mental disorders and produce psychopathology and psychological distress to a high degree (Sateia, 2009). The urgency of treating sleep problems gets visible as they are present in affective, anxiety, eating, pervasive developmental, borderline and antisocial personality disorders, and schizophrenia (Baglioni et al., 2016). In PTSD, studies indicate that sleep mediates the relationship between rumination and more severe PTSD and depressive symptoms (Borders et al., 2015). That is in line with results suggesting that sleep problems hinder the recovery from PTSD (Babson and Feldner, 2010) and that remaining sleep problems after treating PTSD predicted worse treatment outcomes (López et al., 2017). Moreover, non-medical treatment of sleep problems can be as effective as the medical equivalent and can further elevate the effects of treatment for PTSD (Gilbert et al., 2015). Sleep seems to play a substantial role in not only the recovery from psychiatric disorders but also in the recovery from critical illnesses demanding ICU treatment (McKinley et al., 2013). Given that sleep is also correlated with quality of life, this further points to the direction that improving sleep quality could help any patient improve their overall mental state (Zeitlhofer et al., 2000; Košćec Bjelajac et al., 2020).

Limitations of the LD and sleepcoaching approach are that they need further empirical proof. Even though sleepcoaching has been proved useful in shift workers (Holzinger et al., 2019a) and healthy subjects (Holzinger et al., 2019a), it should be further researched in the context of other sleep and mental health problems. We suggest that patient groups with sleep problems like narcolepsy and obstructive sleep apnea would benefit from the program. Furthermore, it should be assessed in the context of other mental health problems involving nightmare and sleep symptoms, such as depression, borderline, and eating disorders. Another limitation to this technique is that LD is not always easy to induce, and empirical evaluations of the induction methods are sparse (Stumbrys et al., 2012). Nevertheless, cognitive techniques combined with wake-up-back-to-bed methods seem promising (Stumbrys et al., 2012), as well as portable devices (Mota-Rolim et al., 2019) and/or support through substances like glantamine (LaBerge et al., 2018).

In conclusion, since sleep problems trigger mental disorders and mental health in general, this may negatively affect recovery from traumas, such as PTSD and other physical illnesses. Nightmares, in turn, have an impact on sleep quality, and they are an indication of psychopathologic underpinnings, including suicide risk. The latter relationship is particularly of interest since this relationship seems to be unidirectional. There is evidence that treating nightmares by LD training programs is effective, as the dreamer might also gain insight into his/her psyche without the disadvantages of long-lasting drug treatments. Applying this approach by including the holistic approach of sleepcoaching into treatment concepts seems especially promising. Sleepcoaching targets various sleep problems; it enables the affected to make informed improvements in their sleep hygiene through education, uses concepts of CBT-I and relaxation techniques, including medical hypnosis, and incorporates dream work through LD techniques.

## Data Availability Statement

The data analyzed in this study is subject to the following licenses/restrictions: Psychtherapeutic Confidentiality. Requests to access these datasets should be directed to Brigitte Holzinger, info@schlafcoaching.org.

## Ethics Statement

The studies involving human participants were reviewed and approved by Ethics Committee MedUni Vienna. The patients/participants provided their written informed consent to participate in this study. Written informed consent was obtained from the individual for the publication of any potentially identifiable images or data included in this article.

## Author Contributions

BH: conceptualization, manuscript, supervision, and LD-training. FN: manuscript, research, and formatting. GK: supervision. All authors contributed to the article and approved the submitted version.

## Conflict of Interest

The authors declare that the research was conducted in the absence of any commercial or financial relationships that could be construed as a potential conflict of interest.

## Publisher's Note

All claims expressed in this article are solely those of the authors and do not necessarily represent those of their affiliated organizations, or those of the publisher, the editors and the reviewers. Any product that may be evaluated in this article, or claim that may be made by its manufacturer, is not guaranteed or endorsed by the publisher.

## References

[B1] American Academy of Sleep Medicine (2014). The International Classification of Sleep Disorders:(ICSD-3). American Academy of Sleep Medicine.

[B2] Babson K. A. Feldner M. T. (2010). Temporal relations between sleep problems and both traumatic event exposure and PTSD: a critical review of the empirical literature. J. Anxiety Disord. 24, 1–15. 10.1016/j.janxdis.2009.08.002 19716676 PMC2795058

[B3] Baglioni C. Altena E. Bjorvatn B. Blom K. Bothelius K. Devoto A.. (2020). The European Academy for Cognitive Behavioural Therapy for Insomnia: an initiative of the European Insomnia Network to promote implementation and dissemination of treatment. J. Sleep Res. 29:e12967. 10.1111/jsr.12967 31856367

[B4] Baglioni C. Nanovska S. Regen W. Spiegelhalder K. Feige B. Nissen C.. (2016). Sleep and mental disorders: a meta-analysis of polysomnographic research. Psychol. Bull. 142, 969–990. 10.1037/bul0000053 27416139 PMC5110386

[B5] Ballesio A. Aquino M. R. J. V. Feige B. Johann A. F. Kyle S. D. Spiegelhalder K.. (2018). The effectiveness of behavioural and cognitive behavioural therapies for insomnia on depressive and fatigue symptoms: a systematic review and network meta-analysis. Sleep Med. Rev. 37, 114–129. 10.1016/j.smrv.2017.01.006 28619248

[B6] Borders A. Rothman D. J. McAndrew L. M. (2015). Sleep problems may mediate associations between rumination and PTSD and depressive symptoms among OIF/OEF veterans. Psychol. Trauma Theory Res. Pract. Policy 7:76. 10.1037/a0036937 25793596

[B7] Buysse D. J. Reynolds I. I. I. C. F Monk T. H. Berman S. R. Kupfer D. J. (1989). The Pittsburgh Sleep Quality Index: a new instrument for psychiatric practice and research. Psychiatry Res. 28, 193–213. 10.1016/0165-1781(89)90047-4 2748771

[B8] Chen P.-H. Kuo H.-Y. Chueh K.-H. (2010). Sleep hygiene education: efficacy on sleep quality in working women. J. Nurs. Res. 18, 283–289. 10.1097/JNR.0b013e3181fbe3fd 21139448

[B9] de Macêdo T. C. F. Ferreira G. H. de Almondes K. M. Kirov R. Mota-Rolim S. A. (2019). My dream, my rules: can lucid dreaming treat nightmares? Front. Psychol. 10:2618. 10.3389/fpsyg.2019.02618 31849749 PMC6902039

[B10] de Sousa I. C. Araújo J. F. de Azevedo C. V. M. (2007). The effect of a sleep hygiene education program on the sleep?wake cycle of Brazilian adolescent students. Sleep Biol. Rhythms 5, 251–258. 10.1111/j.1479-8425.2007.00318.x

[B11] Derogatis L. R. (1977). SCL-90R (Revised Version) Manual I. Baltimore: Johns Hopkins University School of Medicine.

[B12] Ferrans C. E. Powers M. J. (1985). Quality of life index: development and psychometric properties. Adv. Nurs. Sci. 8, 15–24. 10.1097/00012272-198510000-00005 3933411

[B13] Foa E. B. Riggs D. S. Dancu C. V. Rothbaum B. O. (1993). Reliability and validity of a brief instrument for assessing post-traumatic stress disorder. J. Trauma. Stress 6, 459–473. 10.1002/jts.2490060405

[B14] Friedman M. J. (2013). PTSD: pharmacotherapeutic approaches. Focus 11, 315–320. 10.1176/appi.focus.11.3.315

[B15] Gilbert K. S. Kark S. M. Gehrman P. Bogdanova Y. (2015). Sleep disturbances, TBI and PTSD: implications for treatment and recovery. Clin. Psychol. Rev. 40, 195–212. 10.1016/j.cpr.2015.05.008 26164549 PMC5153364

[B16] Görtelmeyer R. (1986). Schlaffragebogen A und B. Weinheim: Internationale Skalen Für Psychiatrie, Beltz.

[B17] Hochard K. D. Heym N. Townsend E. (2015). The unidirectional relationship of nightmares on self-harmful thoughts and behaviors. Dreaming 25:44. 10.1037/a0038617

[B18] Holzinger B. (2008). Kognition im Schlaf (luzides Träumen). Eine Therapiemethode zur Bewältigung von Albträumen - auch bei Traumatisierung, in Gestalt-Traumatherapie, eds Anger H. Schulthess P. (Bergisch-Gladbach: EHP - Verlag Andreas Kohlhage), 223–238.

[B19] Holzinger B. Klösch G. (2013). Schlafcoaching: Wer Wach Sein Will, Muss Schlafen. Wien: Goldegg Verlag.

[B20] Holzinger B. LaBerge S. Levitan L. (2006). Psychophysiological correlates of lucid dreaming. Dreaming 16, 88–95. 10.1037/1053-0797.16.2.88

[B21] Holzinger B. Levec K. Munzinger M.-M. Mayer L. Klösch G. (2019a). Managing daytime sleepiness with the help of sleepcoaching, a non-pharmacological treatment of non-restorative sleep. Sleep Breath. 24, 1–6. 10.1007/s11325-019-01995-0 31853836 PMC7127987

[B22] Holzinger B. Mayer L. Levec K. Munzinger M.-M. Klösch G. (2019b). Sleep coaching: non-pharmacological treatment of non-restorative sleep in Austrian railway shift workers. Arch. Industr. Hyg. Toxicol. 70, 186–193. 10.2478/aiht-2019-70-3244 32597126

[B23] Holzinger B. Saletu B. Klösch G. (2020). Cognitions in sleep: lucid dreaming as an intervention for nightmares in patients with posttraumatic stress disorder. Front. Psychol. 11:1826. 10.3389/fpsyg.2020.01826 32973600 PMC7471655

[B24] Horowitz M. Wilner N. Alvarez W. (1979). Impact of event scale: a measure of subjective stress. Psychosom. Med. 41, 209–218. 10.1097/00006842-197905000-00004 472086

[B25] Hurd R. Bulkeley K. (2014). Lucid Dreaming: New Perspectives on Consciousness in Sleep [2 Volumes]: New Perspectives on Consciousness in Sleep. Santa Barbara: ABC-CLIO.

[B26] Irish L. A. Kline C. E. Gunn H. E. Buysse D. J. Hall M. H. (2015). The role of sleep hygiene in promoting public health: a review of empirical evidence. Sleep Med. Rev. 22, 23–36. 10.1016/j.smrv.2014.10.001 25454674 PMC4400203

[B27] Johns M. W. (1991). A new method for measuring daytime sleepiness: the Epworth sleepiness scale. Sleep 14, 540–545. 10.1093/sleep/14.6.540 1798888

[B28] Košćec Bjelajac A. Holzinger B. Despot Lučanin J. Delale E. A. Lučanin D. (2020). Sleep quality and daytime functioning in older European adults. Eur. Psychol. 25, 186–199. 10.1027/1016-9040/a000406

[B29] Köthe M. Pietrowsky R. (2001). Behavioral effects of nightmares and their correlations to personality patterns. Dreaming 11, 43–52. 10.1023/A:1009468517557

[B30] Krakow B. Schrader R. Tandberg D. Hollifield M. Koss M. P. Yau C. L.. (2002). Nightmare frequency in sexual assault survivors with PTSD. J. Anxiety Disord. 16, 175–190. 10.1016/S0887-6185(02)00093-2 12194543

[B31] LaBerge S. LaMarca K. Baird B. (2018). Pre-sleep treatment with galantamine stimulates lucid dreaming: a double-blind, placebo-controlled, crossover study. PLoS ONE. 13:e0201246. 10.1371/journal.pone.0201246 30089135 PMC6082533

[B32] Lemyre A. Bastien C. Vallières A. (2019). Nightmares in mental disorders: a review. Dreaming 29, 144–166. 10.1037/drm0000103

[B33] Levin R. Nielsen T. A. (2007). Disturbed dreaming, posttraumatic stress disorder, and affect distress: a review and neurocognitive model. Psychol. Bull. 133, 482–528. 10.1037/0033-2909.133.3.482 17469988

[B34] López C. M. Lancaster C. L. Gros D. F. Acierno R. (2017). Residual sleep problems predict reduced response to prolonged exposure among veterans with PTSD. J. Psychopathol. Behav. Assess. 39, 755–763. 10.1007/s10862-017-9618-6 29225414 PMC5716480

[B35] McKinley S. Fien M. Elliott R. Elliott D. (2013). Sleep and psychological health during early recovery from critical illness: an observational study. J. Psychosom. Res. 75, 539–545. 10.1016/j.jpsychores.2013.09.007 24290043

[B36] Morin C. M. (2015). Cognitive behavioral therapy for chronic insomnia: state of the science versus current clinical practices. Ann. Intern. Med. 163, 236–237. 10.7326/M15-1246 26052868

[B37] Mota-Rolim S. A. Pavlou A. Nascimento G. C. Fontenele-Araujo J. Ribeiro S. (2019). Portable devices to induce lucid dreams—are they reliable? Front. Neurosci. 13:428. 10.3389/fnins.2019.00428 31133778 PMC6517539

[B38] Nadorff M. R. Anestis M. D. Nazem S. Claire Harris H. Samuel Winer E. (2014). Sleep disorders and the interpersonal–psychological theory of suicide: Independent pathways to suicidality? J. Affect. Disord. 152–154, 505–512. 10.1016/j.jad.2013.10.011 24182416

[B39] Nadorff M. R. Nazem S. Fiske A. (2011). Insomnia symptoms, nightmares, and suicidal ideation in a college student sample. Sleep 34, 93–98. 10.1093/sleep/34.1.93 21203379 PMC3001802

[B40] Nadorff M. R. Nazem S. Fiske A. (2013). Insomnia symptoms, nightmares, and suicide risk: duration of sleep disturbance matters. Suicide Life Threat. Behav. 43, 139–149. 10.1111/sltb.12003 23278677 PMC3609914

[B41] O'Donnell S. Driller M. W. (2017). Sleep-hygiene education improves sleep indices in elite female athletes. Int. J. Exerc. Sci. 10, 522–530. 28674597 10.70252/DNOL2901 PMC5466408

[B42] Rosenberg R. S. Van Hout S. (2014). The American Academy of Sleep Medicine inter-scorer reliability program: respiratory events. J. Clin. Sleep Med. 10, 447–454. 10.5664/jcsm.3630 24733993 PMC3960390

[B43] Sateia M. J. (2009). Update on sleep and psychiatric disorders. Chest 135, 1370–1379. 10.1378/chest.08-1834 19420207

[B44] Schredl M. (2003). Effects of state and trait factorson nightmare frequency. Eur. Arch. Psychiatry Clin. Neurosci. 253, 241–247. 10.1007/s00406-003-0438-1 14504993

[B45] Schredl M. Dehmlow L. Schmitt J. (2016). Interest in information about nightmares in patients with sleep disorders. J. Clin. Sleep Med. 12, 973–977. 10.5664/jcsm.5928 27070249 PMC4918998

[B46] Sjöström N. Hetta J. Waern M. (2009). Persistent nightmares are associated with repeat suicide attempt. Psychiatry Res. 170, 208–211. 10.1016/j.psychres.2008.09.006 19900715

[B47] Stumbrys T. Erlacher D. Schädlich M. Schredl M. (2012). Induction of lucid dreams: a systematic review of evidence. Conscious. Cogn. 21, 1456–1475. 10.1016/j.concog.2012.07.003 22841958

[B48] Tanskanen A. Tuomilehto J. Viinamäki H. Vartiainen E. Lehtonen J. Puska P. (2001). Nightmares as predictors of suicide. Sleep 24, 845–848. 10.1093/sleep/24.7.845 11683487

[B49] Walters A. S. LeBrocq C. Dhar A. Hening W. Rosen R. Allen R.. (2003). International Restless Legs Syndrome Study Group: validation of the International Restless Legs Syndrome Study Group rating scale for restless legs syndrome. Sleep Med. 4, 121–132. 10.1016/S1389-9457(02)00258-7 14592342

[B50] World Health Organization (Ed.). (2017). ICD-10 Internationale Statistische Klassifikation der Krankheiten und Verwandter Gesundheitsprobleme 10. Revision –BMGF-Version 2017.

[B51] Zeitlhofer J. Schmeiser-Rieder A. Tribl G. Rosenberger A. Bolitschek J. Kapfhammer G.. (2000). Sleep and quality of life in the Austrian population: sleep and quality of life. Acta Neurol. Scand. 102, 249–257. 10.1034/j.1600-0404.2000.102004249.x 11071111

[B52] Zung W. W. (1965). A self-rating depression scale. Arch. Gen. Psychiatry 12, 63–70. 10.1001/archpsyc.1965.01720310065008 14221692

[B53] Zung W. W. (1971). A rating instrument for anxiety disorders. Psychosomat. J. Consult. Liaison Psychiatry 12, 371–379. 10.1016/S0033-3182(71)71479-0 5172928

